# Exploring Perceived Barriers to Physical Activity among Older Adults Living in Low-Population Density Regions: Gender Differences and Associations with Activity Dimensions

**DOI:** 10.3390/healthcare11222948

**Published:** 2023-11-11

**Authors:** María Rúa-Alonso, Antonio Bovolini, Ana Raquel Costa-Brito, Cláudia Vaz, Ermelinda Marques, Nuno Serra, Vítor P. Lopes, Carolina Vila-Chã

**Affiliations:** 1 Polytechnic of Guarda, 6300-559 Guarda, Portugal; bovolini@ipg.pt (A.B.); raquelbrito@ipg.pt (A.R.C.-B.); claudiavaz@ipg.pt (C.V.); emarques@ipg.pt (E.M.); nserra@ipg.pt (N.S.); 2 Research Center in Sports Sciences, Health Sciences, and Human Development (CIDESD), 5001-801 Vila Real, Portugal; vplopes@ipb.pt; 3 Performance and Health Group, Department of Physical Education and Sport, Faculty of Sports Sciences and Physical Education, University of A Coruna, 15179 A Coruña, Spain; 4 Center for Health Technology and Services Research (CINTESIS), 4200-450 Porto, Portugal; 5 Clinical Academic Center of Beiras (CACB), 6200-506 Guarda, Portugal; 6 Polytechnic Institute of Bragança, 5300-223 Bragança, Portugal

**Keywords:** active ageing, YPAS, sedentary behaviour, moving index, vigorous index, health

## Abstract

Older people in low-population density regions tend to have fewer resources to engage in regular physical activity (PA) compared to their counterparts in urban areas. Moreover, PA assumes different dimensions, and the amount of PA related to each dimension may differ between women and men, predisposing them to different PA practices. Therefore, this cross-sectional study aims to describe the prevalence of barriers to PA, gender differences, and their associations with different PA dimensions. A total of 259 older adults (153 women and 106 men; age, 75.17 ± 8.05 years old) living in the community in the region of Guarda (Portugal) were interviewed face to face to record their sociodemographic characteristics, general health status (comorbidity index and self-reported health), PA behaviour, and barriers to PA. Women were more likely to report “low” income and living alone (*p* ≤ 0.05), while men reported a higher negative health status than women (*p* < 0.05). Two intrinsic (“Fear of injury” (40.1%) and “Need for rest” (26.3%)) and two extrinsic barriers (“Lack of nearby facilities” (30.5%) and “I don’t have transport” (25.6%)) were the most prevalent. For women, age, self-reported health, comorbidity index, and intrinsic and extrinsic barriers were similarly associated with the different PA dimensions. However, only self-reported health and extrinsic barriers were the variables associated with the different PA dimensions in men. Therefore, strategies to promote active ageing in low-population density regions should be focused on reducing intrinsic and extrinsic barriers based on gender and the PA dimension to be achieved.

## 1. Introduction

Physical activity (PA) promotion and the reduction of sedentary behaviour (SB) are crucial for protection from non-communicable diseases [1,2], cognitive decline [3], and mental illness [4], as well as reducing all-cause mortality [5] and improving health-related quality of life in the general population, including older adults [6,7]. These benefits are mirrored in PA recommendations, indicating that older adults should engage in at least 150–300 min of moderate-intensity aerobic PA, or at least 75–150 min of vigorous-intensity aerobic PA (or an equivalent combination of moderate- and vigorous-intensity activity) throughout the week, muscle-strengthening activities of moderate or higher intensity and involving all major muscle groups two or more days per week, and perform on three or more days per week multicomponent physical activities focusing on functional balance and strength at moderate or higher intensity, to improve functional fitness and prevent falls [8].

Nevertheless, despite the clear benefits of being active, women and men from all over the world become less active as they get older and do not meet the PA and SB recommendations [9]. Consequently, the promotion of PA in this age group has become a priority in healthy ageing policies [10]. However, to design successful strategies and programmes to promote PA, it is necessary to investigate and understand the perceived barriers experienced by older adults that contribute to decreased PA levels. Some studies have shown that lack of knowledge, skills, abilities, family support, family roles, perceived fears of PA (e.g., pain, injury, or risk of falls) and environmental barriers (e.g., access to facilities and transport or adverse climate conditions) are the main barriers to PA among community-dwelling older adults [11,12,13,14]. However, several factors, such as gender, age, or socioeconomic conditions, may shape how perceived barriers affect SB and PA levels [12,14,15,16]. For instance, previous studies showed gender differences [17,18], with older women reporting a higher prevalence of barriers to moderate to vigorous PA (MVPA) than older men. However, older women are typically more involved in household activities [19], and thus tend to participate more in light-intensity PA (LPA) [20,21]. Along this line, and in relation to LPA, a study conducted by Stalling et al. observed that women spent more time on active transport, home-based activities, and housework than men, while men allocated more time to leisure activities [22].

In view of the different patterns of PA and SB in older adults, it is important to identify the different barriers that older adults may perceive for each of the different PA domains, including the aforementioned factors of gender or age, in order to enable them to comply with the current recommendations of international organisations (i.e., reduce sedentary time to eight hours or less per day and replace it with physically-active behaviours, starting with LPA and progressively introducing MVPA [23]). In this context, LPA has emerged in recent years as a potentially effective strategy for older adults, given its plausibility and the benefits associated with its practice [21,24,25,26,27,28,29]. According to the results obtained by Dupré et al. [29], LPA should be included in future guidelines, since it allows minimum doses to be reached more easily and reduces SB in older adults, and, as their results suggest, the dose–effect curve may be stronger for LPA. 

In this sense, the role of perceived barriers in predicting PA behaviour in older adults remains understudied, especially barriers related to LPA, as most research has investigated the barriers to MVPA [30,31]. This is even more critical in rural and inland areas, which are typically the most neglected and under-resourced regions. As recently stated by Rai et al., the different domains of PA ought to be examined to determine and identify any relationships that occur at that level [17], as, despite the potential of LPA, previous studies did not consider them [14]. 

Therefore, since public strategies to overcome barriers to PA need to be tailored to the specific characteristics and demands of the target population, we aimed to investigate the prevalence and gender differences of perceived barriers to PA among older adults living in low-population density areas, and their association with different dimensions of PA.

## 2. Materials and Methods

Data from the Gmove+ project were used for the analysis. Potential participants were recruited through a dissemination made at public health centres in collaboration with doctors and health professionals, as well as through poster announcements in public and commercial spaces, churches, and social networks in the district of Guarda (Portugal). Data collection was performed by a trained research team, tutored by the principal researcher of the project.

### 2.1. Participants

A total of 259 cases (153 women and 106 men) were included. Participants were recruited from the Guarda health care unit, the day care centres of the district, the physical exercise programmes of the municipality of Guarda, and through the dissemination and promotion of PA activities related to the project.

Exclusion criteria were: (i) aged under 65 years old; (ii) having a mobility impairment; (iii) having cognitive limitations that affect the comprehension or performance of the psychometric tests; and (iv) living in social and health care support centres. All participants were informed about the purpose of the project and signed an informed consent form prior to data collection. The Gmove+ project was approved by the Ethics Committee of the Local Health Unit of Guarda (Ref. 11136), in accordance with the Declaration of Helsinki.

### 2.2. Sociodemographic Characteristics

Individual sociodemographic factors obtained from the face to face interview included: age, gender, education level according to the Portuguese Educational System [32] (<4th Grade, =4th Grade, and >4th Grade), income level (based on the Portuguese average salary scales and grouped into three subcategories: Low (<500 €), Average (500–750 €), and High (>750 €) income, and living arrangement (coded as Living with a partner or Living alone).

### 2.3. General Health Status

The Charlson comorbidity index was used to convert the comorbidities into a score ranging from 0 to 10 [33]. Additionally, the SF-36 questionnaire question “In general, how would you describe your health?” was used to assess self-reported health status [34]. Based on the self-reported health status, participants were classified as having a negative or positive health perception.

### 2.4. Yale Physical Activity Survey

The PA assessment was conducted using the Portuguese version of the Yale PA Survey (YPAS-PT) for older adults [35]. The YPAS-PT determines the type, amount, and pattern of PA for a typical week in the last month. Five activity dimensions (vigorous activity, leisurely walking, moving, standing, and sitting) were obtained by multiplying the partial scores obtained from the questionnaire items (resulting from the multiplication of a frequency score by a duration score for each dimension) by a weighting factor based on the relative intensity of each activity dimension [36,37]. The validity and reliability of this questionnaire have been previously reported [35].

### 2.5. Perceived Barriers to the PA Questionnaire

A Portuguese version of the Perceived Barriers to PA Questionnaire was applied to measure barriers to PA in older populations [38,39]. This 22-item survey employs a five-point Likert scale (“never”, “rarely”, “sometimes”, “often”, and “always”) to rate the frequency with which factors (barriers) interfere with PA practice decisions.

Based on the social-ecological model of recreational PA [40], additional questions about PA program facilities, public transportation, family, and professional assistance were added to the previous questionnaire to better reflect the Gmove+ project’s goal of promoting and disseminating PA programs in the community. Given the importance of introducing new items tailored to local sociocultural contexts, the content and face validity of the questionnaire was initially assessed by our research team, and questions were discussed with a sample of older adults before data collection. Also, standard steps for the adaptation of cultural psychometric scales were followed [41], and questionnaire adjustments were made to improve the participants’ comprehension. The final version of the questionnaire consisted of 29 items. The interviewers were previously trained to gather the information correctly.

### 2.6. Statistical Analysis

Descriptive statistics are shown as means ± standard deviation or as percentages (frequencies), and normality was tested using the Shapiro–Wilk test. Perceived barriers to PA latent factor validity were psychometrically tested, performing a Confirmatory Factor Analysis. Full Information robust Maximum Likelihood was used to handle the small amount of missing data at the item level (missing at random = 3%) as proposed by a previous study [42]. The hypothesized model was tested using Amos 23.0 following the previous recommendations [43]. The absolute fit of the models was evaluated using the chi-square by degrees of freedom ratio (χ^2^/df), and the Standardized Root Mean Square Residual (SRMSR), while the relative fit was assessed using the Normed Fit Index, the Tucker–Lewis Index, and the Comparative Fit Index. For these indices, values over 0.95 indicate a good fit, and values over 0.98 of a very good fit [44]. A value lower than 0.08 for SRMSR is considered acceptable [45]. The Root Mean Square Error of Approximation (RMSEA) was used for evaluating how well the implied model reproduced the variance-covariance matrix of the data, keeping in mind that RMSEA values as low as 0.08 are deemed adequate, and below 0.06 represent a good fit to the model [45]. Then, the extracted factors were used in a multiple regression analysis with stepwise variable selection to identify the PA predictors [42].

For gender comparisons, Pearson’s chi-squared (χ^2^), an independent sample *t* test, or the Mann–Whitney *U* test, respectively, were used. Moreover, stepwise regression analysis was performed. The PA dimensions (vigorous, leisurely walking, moving, standing, and sitting) were treated as dependent variables, while the intrinsic and extrinsic barriers to PA, age, comorbidities index and self-reported health status were treated as independent variables. In all regression models checked, the collinearity was evaluated by the Variance Inflation Factors test, and the goodness of fit model was verified by the Nagelkerke R square test. IBM SPSS Statistics v.24.0 (IBM Corp, Armonk, NY, USA) was used for statistical analysis and the statistical significance level was set at 0.05.

## 3. Results

**Figure 1:**
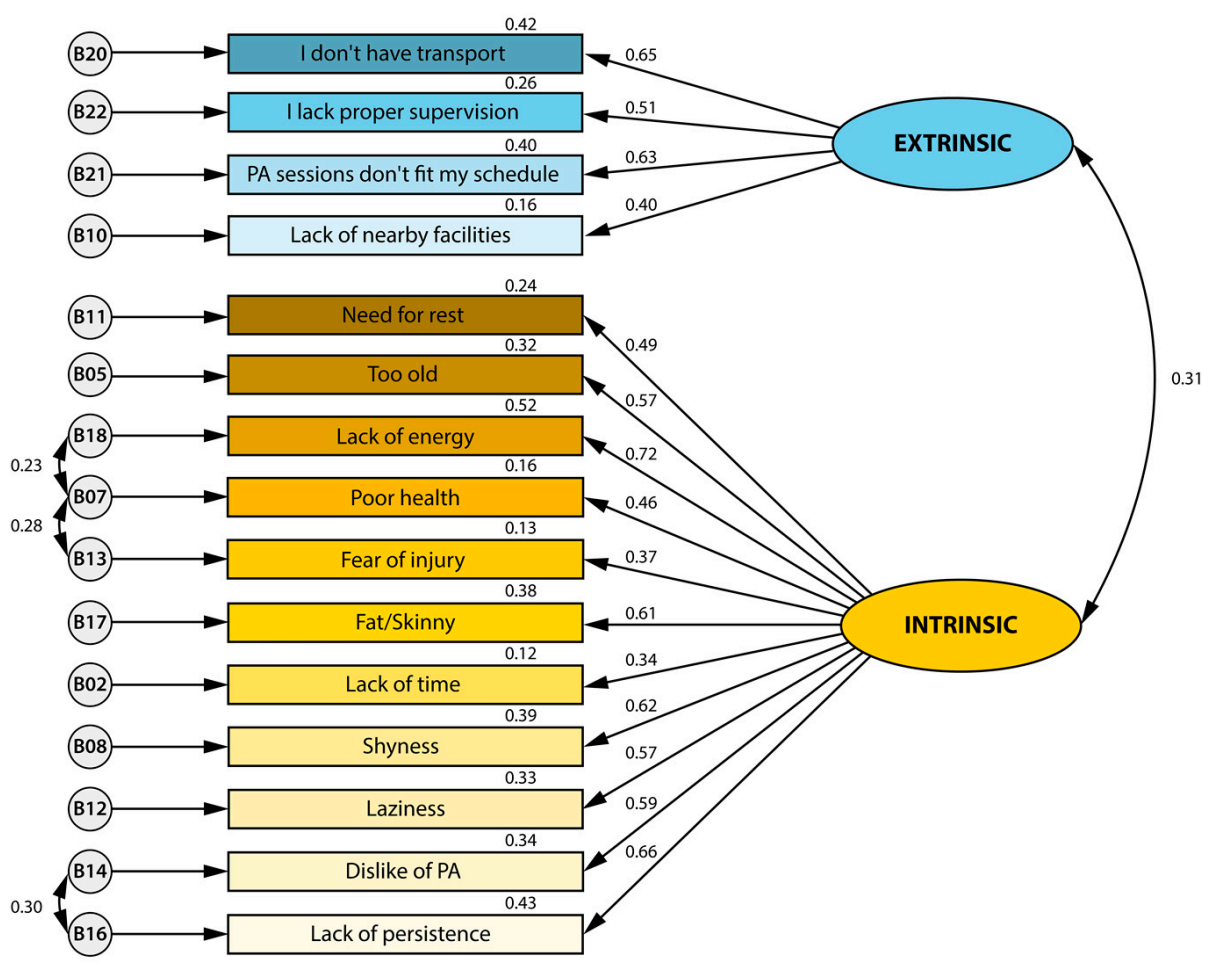
Theoretical model of barriers to Physical Activity Questionnaire Portuguese version with the factor loading in each dimension.

Table 1 and Figure 1 present the results of the confirmatory factor analysis model used to confirm the questionnaire-based factors for perceived barriers to PA. Results from this model indicate that the index factor analysis fit the model well with the 2-factor structure and 15 of 29 items. The existence of intrinsic and extrinsic factors was considered based on the meaning of the items that compose each factor. On the main fit measures, the model showed appropriate scores.

Sociodemographics, health status, and indicators of PA levels are summarised in Table 2. Men and women were matched for age, with 70–79 years being the most frequent age range (43.1%). Most participants had completed the 4th grade of schooling, regardless of gender. Women were more likely to report “Low income” (45.8% vs. 28.3%) and to live alone (49.7% vs. 32.1%) than men (*p* ≤ 0.05). Regarding general health status, no gender differences were present in the comorbidity index. Both women (92.7%) and men (74.5%) claimed to have a positive self-reported health status. However, male participants reported a higher negative health status than women (*p* < 0.05). No gender differences were found in the PA dimensions.

**Figure 2:**
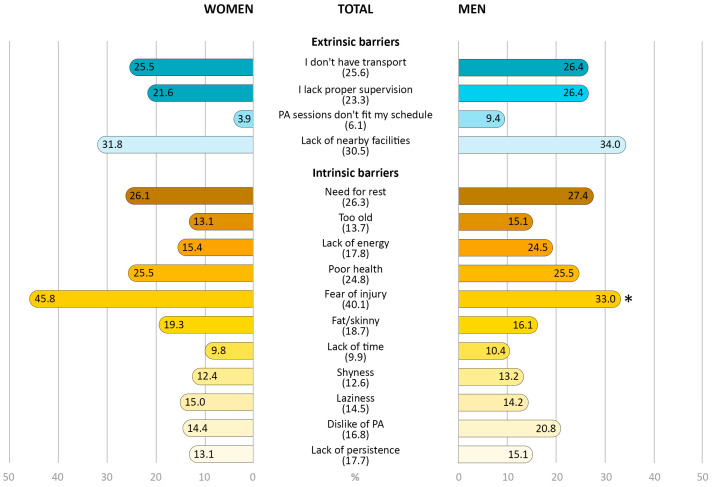
Prevalence of total and gender-specific perceived barriers to PA. Extrinsic barriers are represented in blue, and intrinsic barriers in yellow. * differences in gender distribution. Data are presented as percentages (%) (*n* = 259).

Results of the perceived barriers to PA questionnaire are presented in Figure 2. The results showed that “Fear of injury (40.1%), “Lack of nearby facilities” (30.5%), “Need for rest” (26.3%), and “I don’t have transport” (25.6%) were the most prevalent barriers to PA among all participants. Women presented similar values compared to men except for the barrier “Fear of injury” (45.8% vs. 33.0%; *p* < 0.01), reporting a higher percentage than men.

Table 3 and Table 4 show the associations between the PA dimensions, intrinsic and extrinsic barriers to PA, health status, and age for women and men, respectively. For women (Table 3), model 2, which included the comorbidity index and self-reported health status, better explained (7%) the variance of vigorous index [F (1.150) = 6.390; R^2^ = 0.068; *p* = 0.002]. Meanwhile, the intrinsic factors (model 1) were the only factor with explanatory power (6%) for the leisure walk index [F (1.152) = 11.042; R^2^ = 0.062; *p* = 0.001]. Associations with moving index were suggested by three models, with model 3 (age, intrinsic factors, and extrinsic factors) demonstrating the best explanatory power [F (3.149) = 5.673; R^2^ = 0.084; *p* = 0.001]. Only two models suggested significant associations with the standing index, with model 2 having the best explanatory power [F (2.152) = 6.260; R^2^ = 0.065; *p* = 0.002]. In the associations with the sitting index, only age (model 1) showed a significant association with an explanatory power of 9% [F (1.152) = 16.552; R^2^ = 0.093; *p* < 0.001]. For men (Table 4), self-reported health status alone explains 3% of the vigorous index variance [F (1.99) = 4.365; R^2^ = 0.033; *p* = 0.039], 12% of the leisure walking index variance [F (1.100) = 14.230; R^2^ = 0.116; *p* < 0.001], 3% of the standing index variance [F (1.100) = 4.213; R^2^ = 0.031; *p* = 0.043], and 3% of the sitting index [F (1.100) = 2.936; R^2^ = 0.034; *p* = 0.034]. However, in the associations with the moving index, extrinsic factors presented 10% of explanatory power [F (1.100) = 11.531; R^2^ = 0.094; *p* = 0.001].

## 4. Discussion

The main findings of this study are that (i) two intrinsic (“Fear of injury” and “Need for rest”) and two extrinsic barriers (“Lack of nearby facilities” and “I don’t have transport”) were the most reported, with women being more likely to express “Fear of injury”; (ii) for women, all factors (age, self-reported health status, comorbidity index, and intrinsic and extrinsic barriers) were associated with the different PA dimensions; and (iii) only self-reported health and extrinsic barriers were the factors associated with the different PA dimensions in men.

### 4.1. Prevalence and Gender Differences of Perceived Barriers to PA

Regarding intrinsic barriers, “Fear of injury” is the most frequent barrier to PA among the older adults participating in this study (40.1%), being higher in women. According to the Special Eurobarometer 525 for Sport and Physical Activity, this is the fourth reason among the population aged 55 and over in Europe. In Portugal in particular, this barrier has increased by 10% since 2017. This is probably due to the Portuguese population ageing [46]. Also, at least one in four older people reported that the second reason is “Need to rest” (26.3%), closely followed by “Poor health” (24.8%), which is the most frequently reported according to the Eurobarometer reports in older populations [46]. These data are consistent with previous research [47,48,49] reporting that fear of injury, as well as fear of falling and health problems, are the main barriers in older populations. Indeed, a recent systematic review showed a consistent association between PA with increased fear of falling, but not with falls or fractures [50]. As the authors reinforce, it is necessary to promote PA-related health behaviours, as fear of falling influences mobility, increases social isolation, and decreases levels of self-efficacy, which are other potential barriers to PA and factors related to quality of life. In particular, for the Portuguese population, a recent study investigated PA barriers across multiple age groups and reported that, for older adults, health problems and a dislike of exercise or lack of motivation were the most prevalent [48], partially confirming our findings regarding the main barriers to PA. Moreover, other participant characteristics may explain these findings. For instance, reduced educational levels are likely to lead to lower health literacy, and thus less comprehension of the health benefits of regular PA [14,15,51].

For extrinsic barriers, “Lack of nearby facilities” and “I don’t have transport” emerged as the main barriers to PA. Also, both barriers seem to be closely related. Previous studies in large cities showed how a greater supply of public transport allows people to move around more easily, in contrast to regions with low population density where the transport network is limited [12,14,15], and how the opportunity to practice systematic PA in the countryside is scarcer than in large centres due to the limited amount of infrastructure in the inland population [16]. Indeed, evidence referred to the “decentralisation of facilities” as a crucial facilitator of regular PA practice [12,52]. Furthermore, and in relation to extrinsic barriers, Gray et al. previously observed that socioeconomic status can influence barriers to PA. Those with lower socioeconomic status often report more barriers and, consequently, lower levels of PA practice, which is also influenced by age. [53]. This is partially in line with our results, which show that more than half of our participants have a low income (below the national minimum living wage), which may have an impact on barriers to PA.

Regarding gender differences, although there are some socioeconomic disparities between women and men, our data only report differences for the intrinsic barrier “Fear of injury”. Thus, although socioeconomic status is an independent factor acting as a barrier to PA [47], our sample does not seem to be influenced by it. Contrary to Lee [20], our study did not find that environmental (or extrinsic) factors specific to women represent worse conditions for their PA practice. Similarly, although men were more likely to report negative self-reported health status, our results did not observe differences in the “Poor health” barrier, contrary to previous studies [17,20,54]. Historically, older Portuguese women are less involved in regular PA, exercise, or sports activities [46,55,56], and are more sedentary than men [57,58]. However, perceived PA levels are normally overestimated in older women, largely conditioned by the fact that they are involved in more domestic tasks compared to men [59,60,61]. Likewise, there are gender differences in life trajectories related to social and health aspects [62]. In that sense, regular PA practice in older adults is positively related to education, exercise history, and self-efficacy [63,64], which tends to be lower in women of all ages and has been established as a barrier to PA [53,65]. This may be closely related to fear of injury, being the only barrier that showed gender differences in our sample, in agreement with Arazi et al. [47].

### 4.2. Correlation between PA Barriers and PA Dimensions

In the regression model, the comorbidity index, self-reported health status, and age variables were added to the model, as they showed correlations with intrinsic and extrinsic factors. Indeed, previous studies have reported that age, comorbidities, and self-rated health status were independent barriers to PA [14,49,66]. As initially hypothesised, the health-related factor was associated with different PA dimensions, but this was mainly observed in men. A potential explanation for these results is that they may increase their participation in regular physical activity if they feel good, regardless of age, and illness, among other factors. Unlike men, more factors correlated with different PA dimensions among women. These findings are consistent with previous research, which found that women are more easily affected by sociodemographic and health-related outcomes, resulting in low adherence to PA programmes [14,67].

Consistent with previous findings, which showed that poor health outcomes were positively and independently related to the odds of non-adherence to the recommended levels of MVPA [30], health-related issues were negatively correlated with the vigorous PA dimension in both genders. However, in women, possible comorbidities play a significant role in the vigorous PA engagement in this study. Although both women and men presented a reduced number of factors correlated to the leisure walking domain in our study, there were gender differences in the associations. Our results contradict a previous study that described a higher number of factors associated with leisure walking PA [68]. A possible explanation is that both genders have no difficulty achieving this PA dimension, classified as light–moderate PA, and it is more likely to be part of the daily life of this population. On the other hand, one possible reason for the gender differences in the associated factors for leisure PA is the fact that men tend to report engaging in more exercise for enjoyment than women, potentially because women tend to spend more time on household chores [48]. Extrinsic factors were correlated with the PA enrolled as the Moving index domain in both genders. In this regard, place of residence has been shown to be a determinant factor, notably for older people living in rural areas, where services or support are generally lacking compared to urban areas [41]. For women, in addition to extrinsic barriers, age and intrinsic barriers are other factors that explain the Moving index. Regarding the age factor, older adults are aware of their age-related physical limitations, leading to a lack of confidence in their abilities [30,69]. As noted above, women in this study had a higher prevalence of the “Fear of injury” barrier than men. It, therefore, seems reasonable that these two factors are determinants for our women, and not for their counterparts.

To our knowledge, the relationships between barriers to PA and the standing and sitting indices are the least investigated. Concerning the Standing index, a growing interest has emerged as studies show that increasing the volume of light to moderate PA in older individuals may positively affect their health status [70,71]. Based on our results, improving modifiable environmental conditions to reduce external barriers is a promising strategy to increase this PA dimension in older women. Also, the effect of age can be counteracted with awareness programs on the benefits of regular PA and the adaptation of facilities to the needs of this population. In contrast, self-reported health status was the factor most related to the standing domain in men. In fact, they were significantly more likely to report negative values for this factor. Therefore, modifying their perception of health status seems to be key in men, specifically to promote more time in the standing position and, in general, to increase their PA levels and reduce SB. Finally, sitting is the most common SB among older populations and is related to an increased risk of cardiovascular disease and all-cause mortality [72]. Age and self-reported health status were predictors for sitting time in our female and male participants, respectively. Ageing itself (reported by women) and its characteristics play a key role in the development of SB. Along with declining physical, mental, and cognitive health (reported by men), which limits their capacity and/or motivation for standing for long periods of time or performing PA, older adults describe additional leisure time associated with retirement, mostly occupied with typically sedentary leisure tasks (sewing, playing cards, playing chess, etc.) [73].

### 4.3. Confirmatory Factor Analysis Model

Our results showed that these two barriers are theoretically meaningful for PA engagement in this population. Extrinsic barriers refer to the infrastructure in the neighbourhoods and communities, while intrinsic barriers include personal motivations often associated with health, mood, and psychosocial factors [14,74].

### 4.4. Limitations, Strengths, and Future Directions 

In general, the findings of this study revealed that results for women are consistent with the ecological model [30], which implies that, when compared to men, a greater variety of sociodemographic and health-related characteristics function as predictors of PA behaviours. However, our study presents some limitations. Firstly, results related to physical activity may have been affected by the subjective assessment of PA. This may have magnified or diminished real PA levels compared to more objective measures such as accelerometery. Additionally, the non-consideration of local sociocultural features (such as health literacy) by the assessment tools may also represent a limitation in the collection and interpretation of the results. Strengths of this research include an exploration of associations between LPA, SB, and barriers to PA, as the most recent guidelines recommend examining the factors that influence these PA behaviours, especially in older individuals. Moreover, the study was performed with older adults belonging to low-population density regions, mostly in rural contexts, revealing barriers to PA that were not identified in larger Portuguese studies. Thus, future research is recommended to address the issue of PA barriers using other approaches; to consider not only the barriers to different levels of PA, but also the existence of facilitators and beliefs about the benefits of regular PA, and, in many cases, to perform cross-cultural adaptation even for well-established questionnaires, as suggested by the literature.

### 4.5. Practical Applications

Identifying barriers and facilitators of PA considering geographical context should be the first strategy before developing community PA programs. Previous studies have identified a global trend, but identifying local differences can lead to more tailored and effective policies and guidelines to encourage an active lifestyle. Identifying the most common barriers that inhibit the regular practice of PA is crucial, since evidence suggests that gradually accumulating light to moderate levels of PA may positively impact the general health status of older people.

## 5. Conclusions

The achievement of the recommended levels of PA is influenced by several perceived barriers to PA, among other factors. As demonstrated by our findings, more specifically by the observed associations between barriers and light to moderate levels of PA, educating the population about the health benefits of achieving the recommended levels of PA is a priority. Likewise, decreasing extrinsic barriers (i.e., improving access to facilities and providing public transportation to fitness or sports centres) should be the priority in promoting PA, especially among older women living in low-population density regions. Furthermore, PA decentralisation policies should be implemented as prerequisites to increase population adherence for regular participation in PA.

## Figures and Tables

**Table 1:** Confirmatory factor analysis.

	χ^2^/df	SRMR	NFI	CFI	RMSEA
Model	192.84/0.86	0.072	0.810	0.882	0.070

χ^2^/df = Chi-square by degrees of freedom ratio; SRMR = Root mean square residual; NFI = normed fit index; CFI = comparative fit index; RMSA = root mean square error of approximation.

**Table 2:** Sociodemographic, general health status, and levels of PA characteristics of the participants.

Characteristics	Total Sample (*n* = 259)	Women (*n* = 153)	Men (*n* = 106)
**Sociodemographic**			
*Age (years)* ^¥^	75.17 ± 8.05	75.14 ± 7.96	75.22 ± 8.22
60–69	27.5 (72)	26.8 (41)	29.2 (31)
70–79	43.1 (113)	45.1 (69)	41.5 (44)
≥80	28.2 (74)	28.1 (43)	29.2 (31)
*Education level* ^¥^			
<4th Grade	17.9 (47)	19.6 (30)	16.0 (17)
4th Grade	52.7 (138)	53.6 (82)	52.8 (56)
>4th Grade	28.2 (74)	26.8 (41)	31.1 (33)
*Income level* ^¥^			
Low (<500€)	38.2 (100)	45.8 (70)	28.3 (30) *
Average (500–750€)	16.4 (43)	15.0 (23)	18.9 (20)
High (>750€)	44.3 (116)	39.8 (60)	52.8 (56)
*Living arrangement* ^¥^			
Living with a partner	56.9 (139)	50.3 (77)	67.9 (72)
Living alone	43.1 (120)	49.7 (76)	32.1 (34) *
**General health Status**			
*Comorbidity index* ^§^	3.33 (0.57)	4.71 (1.37)	4.47 (1.30)
*Self-reported health status* ^¥^			
Positive	81.2 (211)	92.7 (139)	74.5 (79)
Negative	18.8 (48)	7.3 (14)	25.5 (27) *
**PA dimensions index**			
Vigorous index (u/m)	16.66 ± 5.57	8.82 ± 9.64	8.82 ± 6.78
Walking index (u/m)	13.33 ± 4.61	13.91 ± 7.98	13.98 ± 9.07
Moving index (h/d)	11.11 ± 4.58	9.37 ± 3.28	8.39 ± 3.54
Standing index (h/d)	7.33 ± 3.05	6.06 ± 2.10	6.23 ± 2.24
Sitting index (h/d)	2.34 ± 1.86	2.22 ± 0.92	2.15 ± 0.81

Data are presented as means ± SD or as percentages and sample size [% (*n*)]. ^¥^: Chi-squared test was computed to compare the distributions by gender; ^§^: independent sample *t* test or Mann–Whitney *U* test were computed to compare the distributions by gender; * *p* ≤ 0.05. PA = physical activity; u/m = units/month; h/d = hours/day.

**Table 3:** Association between PA dimensions and PA barriers, health status, and age for women (*n* = 153).

Physical Activity Dimension *	R^2^	β	*p*
Vigorous index (units/month)			
*Model 2*|*comorbidities + self-reported health status*	0.07	−0.122	0.002
Leisure walking index (units/month)			
*Model 1*|*Intrinsic factors*	0.06	−0.261	0.001
Moving index (hours/day)			
*Model 3*|*Age + intrinsic factors + extrinsic factors*	0.08	0.182	0.001
Standing index (hours/day)			
*Model 2*|*Age + extrinsic factors*	0.07	−0.158	0.002
Sitting index (hours/day)			
*Model 1*|*Age*	0.09	0.314	<0.001

* Yale Physical Activity Survey; stepwise regression model included age, self-reported health, comorbidity index, and intrinsic and extrinsic barriers.

**Table 4:** Association between PA dimensions and PA barriers, health status, and age for men (*n* = 106).

Physical Activity Dimension *	R^2^	β	*p*
Vigorous index (units/month)			
*Model 1*|*Self-reported health status*	0.03	−0.205	0.039
Leisure walking index (units/month)			
*Model 1*|*Self-reported health status*	0.12	−0.353	<0.001
Moving index (hours/day)			
*Model 1*|*Extrinsic factors*	0.09	−0.322	0.001
Standing index (hours/day)			
*Model 1*|*Self-reported health status*	0.03	−0.201	0.043
Sitting index (hours/day)			
*Model 1*|*Self-reported health status*	0.03	0.209	0.035

* Yale Physical Activity Survey; stepwise regression model included age, self-reported health, comorbidity index, and intrinsic and extrinsic barriers.

## Data Availability

Data are available on request.
